# Nicotinic Acetylcholine Receptor Agonists Attenuate Septic Acute Kidney Injury in Mice by Suppressing Inflammation and Proteasome Activity

**DOI:** 10.1371/journal.pone.0035361

**Published:** 2012-05-07

**Authors:** Prodyot K. Chatterjee, Michael M. Yeboah, Oonagh Dowling, Xiangying Xue, Saul R. Powell, Yousef Al-Abed, Christine N. Metz

**Affiliations:** 1 The Center for Immunology and Inflammation, The Feinstein Institute for Medical Research, Manhasset, New York, United States of America; 2 Renal Division, Brigham and Women’s Hospital, Harvard Medical School, Boston, Massachusetts, United States of America; 3 The Center for Heart and Lung Research, The Feinstein Institute for Medical Research, Manhasset, New York, United States of America; 4 The Center for Biomedical Science, The Feinstein Institute for Medical Research, Manhasset, New York, United States of America; Universidade de Sao Paulo, Brazil

## Abstract

Sepsis is one of the leading causes of acute kidney injury (AKI). Septic patients who develop acute kidney injury (AKI) are at increased risk of death. To date there is no effective treatment for AKI or septic AKI. Based on their anti-inflammatory properties, we examined the effects of nicotinic acetylcholine receptor agonists on renal damage using a mouse model of lipopolysaccharide (LPS)-induced AKI where localized LPS promotes inflammation-mediated kidney damage. Administration of nicotine (1 mg/kg) or GTS-21 (4 mg/kg) significantly abrogated renal leukocyte infiltration (by 40%) and attenuated kidney injury. These renoprotective effects were accompanied by reduced systemic and localized kidney inflammation during LPS-induced AKI. Consistent with these observations, nicotinic agonist treatment significantly decreased renal IκBα degradation and NFκB activation during LPS-induced AKI. Treatment of human kidney cells with nicotinic agonists, an NFκB inhibitor (Bay11), or a proteasome inhibitor (MG132) effectively inhibited their inflammatory responses following stimulation with LPS or TNFα. Renal proteasome activity, a major regulator of NFκB-mediated inflammation, was enhanced by approximately 50% during LPS-induced AKI and elevated proteasome activity was significantly blunted by nicotinic agonist administration *in vivo.* Taken together, our results identify enhanced renal proteasome activity during LPS-induced AKI and the suppression of both proteasome activity and inflammation by nicotinic agonists to attenuate LPS-induced kidney injury.

## Introduction

The kidney is a frequent target for sepsis-associated multi-organ injury, with increasing prevalence of acute kidney injury (AKI) observed among patients with severe sepsis (23%) and septic shock (51%) compared to patients with moderate sepsis (19%) (Reviewed in [1], [2]). Indeed severe sepsis and septic shock are the leading causes of AKI in intensive care patients and may be responsible for more than 50% of cases of AKI in such patients. Septic patients who develop AKI have overall increased morbidity, require prolonged hospital stays, use more healthcare resources, and have increased mortality. The pathophysiology of septic AKI is complex, multi-factorial and distinct from non-septic AKI [2]–[5]. Epidemiological, clinical, and experimental studies support the concept that pro-inflammatory mediators produced during sepsis promote intrarenal hemodynamic changes, endothelial dysfunction, leukocyte infiltration, and damaging tissue inflammation that lead to renal failure. Unfortunately, despite several years of intense investigations and significant advances in medicine and therapeutics, there are still no effective treatments for septic AKI.

Experimental endotoxemia induced by LPS, the major endotoxin of gram negative bacteria, is the most frequently employed model to study septic AKI (LPS-induced AKI). This model produces consistent renal tissue damage that is similar to that observed in humans [6]–[9]. Lipopolysaccharide (LPS) disseminates to the kidney proximal tubules and microvilli of the brush border, and is found associated with epithelial cells and endothelial cells of the peritubular capillaries [10]. Localized LPS promotes TNFα production by resident renal tubular epithelial cells [11], mesangial cells [12], glomerular cells [13], as well as leukocytes which adhere to the endothelium [12]. Thus, renal TNFα is proposed to be responsible for some of the renal dysfunction observed in LPS-induced AKI [6]; [14] through both direct cellular injury [15] and the amplified induction of localized inflammatory mediators (via NFκB activation), including cytokines and chemokines which stimulate the infiltration of damaging neutrophils and monocytes into the kidneys, particularly the renal cortex [6]. In addition, extrarenal and systemic TNFα contributes to kidney injury observed during septic-AKI [7]. Concurrent vascular inflammation and dysfunction characterized by endothelial cell activation, microvascular injury, increased vascular permeability, intraglomerular thrombosis, and reduced renal perfusion are typically observed in sepsis-associated AKI [12], [16].

Because the pathogenesis of sepsis-associated AKI is ultimately mediated through multiple pathways, a multi-targeted therapeutic approach is warranted. One recently described broad-based physiologic mechanism for regulating inflammation is the cholinergic anti-inflammatory pathway [17]. This pathway can be stimulated by the administration of nicotinic acetylcholine receptor (nAChR) agonists, including nicotine and GTS-21, to produce beneficial anti-inflammatory effects [18]–[22], even in the absence of an intact vagus nerve [23]. Recent studies show that nicotinic agonists abrogate renal damage during experimental ischemia-reperfusion injury [23], [24]. While a localized anti-inflammatory effect was observed, the exact mechanism involved in mediating the anti-inflammatory effects of nicotinic agonists is not well understood. We investigated the effect of nicotinic agonists on renal NFκB and proteasome activity, as well as renal damage during septic AKI.

## Results

### Nicotinic Agonists Attenuate LPS-induced AKI

**Figure 1:**
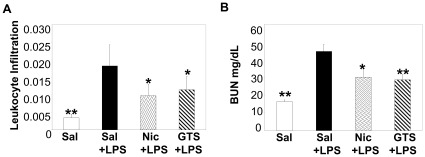
Nicotinic agonists protect against kidney damage during LPS-induced septic AKI. (A-B) Mice were treated with saline alone or LPS (5 mg/kg) plus either saline, nicotine (1 mg/kg), or GTS-21 (4 mg/kg, i.p). (A) Renal leukocyte infiltration was determined by measuring kidney MPO levels by ELISA. (B) BUN levels (mg/dL) were assessed 24 hrs post LPS. Data are shown as mean±SD. *p<0.05, **p<0.001 vs. Sal+LPS-treated mice. n = 5 for saline alone group and n = 8 for all other groups.

Administration of either nicotine or GTS-21 during LPS-induced septic AKI model significantly suppressed kidney injury induced by LPS. Both nicotine and GTS-21 treatment reduced leukocyte infiltration (assessed by renal myeloperoxidase (MPO) levels) within the kidney by approximately 40%, during experimental LPS-AKI compared to saline treatment (Fig. 1A). Consistent with the decline in leukocyte trafficking to the kidneys, improved renal function (as suggested by BUN levels) was observed (Fig. 1B).

### Nicotinic Agonists Regulate Systemic and Renal Inflammation During LPS-induced AKI

**Figure 2:**
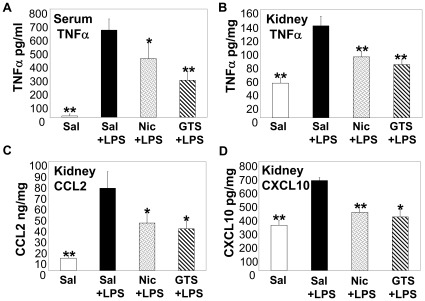
Nicotinic agonist administration reduces systemic and local renal inflammation during septic AKI. Mice were treated with saline alone (Sal, i.p.) or LPS (5 mg/kg, i.p.) plus either saline (i.p.), nicotine (Nic, 1 mg/kg, i.p.), or GTS-21 (GTS, 4 mg/kg, i.p.). Serum TNFαlevels (A) and renal TNFα, CCL2 and CXCL10 levels (B–D) were determined by ELISA. Data are shown as mean±SD, as pg/ml (A) or corrected for protein concentration, pg/mg kidney tissue (B-D). *p<0.05, **p<0.001 vs. Sal+LPS-treated mice. n = 5 for saline alone group and n = 8 for all other groups.

LPS produces renal damage by directly stimulating TNFα production within the kidney [6], [11]–[13] and by increasing circulating TNFα levels [7]. Serum TNFα levels were enhanced during LPS-AKI and treatment with nicotinic agonists reduced serum TNFα levels (Fig. 2A). In addition, septic AKI was accompanied by significant kidney inflammation as measured by kidney TNFα, CCL2, and CXCL10 levels (Figs. 2B–D); renal concentrations of these pro-inflammatory mediators were blunted by treatment with nicotinic agonists (Figs. 2B–D).

### Chemokine Production by Human Kidney Cells is Reduced by Nicotinic Agonists

**Figure 3:**
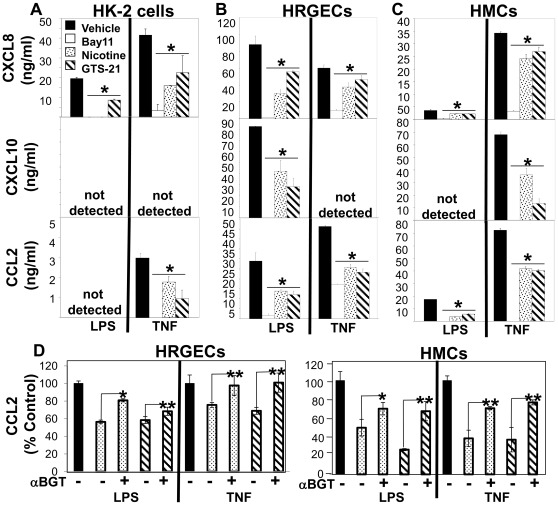
Nicotinic agonists suppress inflammatory responses by human kidney cell populations. (A) Human HK-2 renal tubular epithelial cells, (B) human renal glomerular endothelial cells (HRGECs), and (C) human mesangial cells (HMCs) were treated with either vehicle (solid bars), Bay 11, an NFκB inhibitor (20 µM, open bars), nicotine (10 µM, speckled bars), or GTS-21 (1 µM, hatched bars) and then either LPS (1 µg/ml), or TNFα (20 ng/ml). Supernatants (n = 3 wells per condition) were analyzed for CXCL8 (upper panel), CXCL10 (middle panel), and CCL2 (lower panel) by ELISA. Data are shown as cytokine levels (pg/ml) (mean±SEM) of 3 independent experiments. *p<0.05 vs. LPS- or TNFα-treated control (vehicle) cells. (D) HRGECs and HMCs were treated with saline or αBGT (2 µM) prior to treatment with nicotine (10 µM, speckled bars), or GTS-21 (1 µM, hatched bars), and then either LPS (1 µg/ml) or TNFα (20 ng/ml) (solid bars), as indicated. Supernatants (n = 3 wells per condition) were analyzed for CCL2. Data are shown as % control with 100% saline+LPS or saline+TNF. *p<0.05 and **p<0.01 comparing ±αBGT.

To determine the potential cellular targets of nicotinic agonists within the kidney we examined the effect of nicotine and GTS-21 on both LPS- and TNFα-induced pro-inflammatory mediator production by several kidney cell populations, including human renal proximal tubular epithelial cells (HK-2 cells), human renal glomerular endothelial cells (HRGECs), and human mesangial cells (HMCs) (Fig. 3A–C). Stimulation with LPS induced significant production of CXCL8 by HK-2 cells, HRGECs, and HMCs, CXCL10 by HRGECs, and CCL2 by HRGECs and HMCs (Fig. 3). TNFα treatment induced significant production of both CXCL8 and CCL2 by HK-2 cells, HRGECs, and HMCs, and CXCL10 by HMCs (Fig. 3). Treatment of HK-2 cells with nicotinic agonists significantly reduced both LPS- and TNFα-induced CXCL8 and TNFα-induced CCL2 (Fig. 3A). We observed that treatment of HRGECs with either nicotine or GTS-21 (at optimal concentrations) significantly blocked LPS-induced CXCL8, CXCL10, and CCL2 production (Fig. 3B) and TNFα-induced CXCL8 and CCL2 production (Fig. 3B). Treatment of HMCs with nicotinic agonists significantly suppressed LPS-induced CXCL8 and CCL2 production (Fig. 3C) and TNFα-induced CXCL8, CXCL10, and CCL2 production (Fig. 3C). As expected, Bay 11, a cell-permeable inhibitor of IκBα phosphorylation and hence, inhibitor of NFκB activation, significantly reduced LPS- and TNFα-induced chemokine production by all three cell types (Fig. 3A–C). Treatment of HRGECs and HMCs with αbungarotoxin (αBGT), an α7nAChR selective antagonist, prior to nicotinic agonists partly reversed their inhibitory effects on TNF- and LPS-induced CCL2 production (Fig. 3D).

### Kidney Cells Express α4, α7, and β2 nAChR Subunits

**Figure 4:**
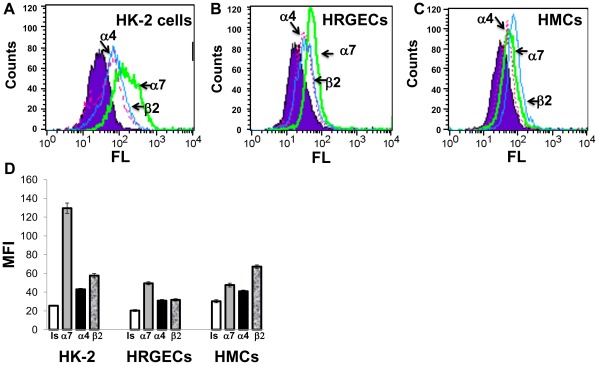
Human Kidney Cells Express nAChR subunits. To assess nAChR protein levels, (A) HK-2, (B) HRGECs, and (C) HMCs were analyzed by flow cytometry using specific antibodies against the α7nAChR (green, thick solid line), α4nAChR (pink, dotted line), and β2nAChR (blue, thin solid line) vs. isotype control (shaded plot). Histogram plots (FL-nAChR subunits vs. counts) are shown for each cell type. (D) Graph shows the average geometric mean MFI (±SD) for each cell type and each nAChR subunit (isotype control = Is; α7nAChR = α7; α4nAChR = α4; and β2nAChR = β2). n = 3 samples per each condition and cell type.

While nicotine is a non-specific nAChR agonist, GTS-21 is a partial selective agonist of the α7nAChR but can also bind to α4β2nAChRs. Using QPCR methods we detected the presence of α7, α4, and β2 nAChR subunits in normal mouse kidneys. When expressed as a ratio of specific nAChR subunit mRNA:HPRT1 (housekeeping gene) mRNA, α4, α7, and β2 nAChR subunit expressions were 2.292±0.21; 1.628±0.054; 1.334±0.091, respectively. We used the human kidney cells/cell lines to explore the expression of these nAChR subunits by various kidney cell types using flow cytometry methods. All three cell types expressed all three nAChR subunits, with α7nAChR expression the highest for both HK-2 (proximal tubule epithelial cells) and HRGECs (renal glomerular endothelial cells) (Fig. 4).

### Nicotinic Agonists Suppress LPS-induced AKI-associated NFκB Activation

**Figure 5:**
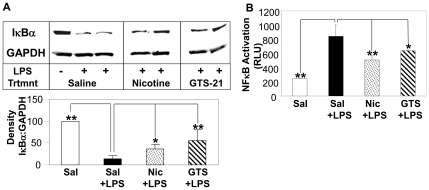
Nicotine and GTS-21 treatment inhibits IκBα degradation and NFκB activation within kidneys during septic AKI. Mice were treated with saline alone (i.p.) or LPS (5 mg/kg, i.p.) plus either saline, nicotine (1 mg/kg, i.p.), or GTS-21 (4 mg/kg, i.p.). Kidney tissues were analyzed for (A) IκBα degradation and (B) NFκB activation. (A) Cytoplasmic IκBα and GAPDH levels were assessed by Western blotting (representative blot is shown) and the density of the bands were analyzed. The data are shown as the ratio of IκBα:GAPDH densities. (B) NFκB activation was determined as described in the Methods section. Data are shown as NFκB activation (expressed as relative light units, RLU)(mean ± SD). *p<0.05, **p<0.001 vs. Sal+LPS. n = 5 for saline alone group and n = 8 for all other groups.

]NFκB activation within the kidney regulates the production of numerous pro-inflammatory mediators during septic AKI [25]. Next, we examined the effects of nicotinic agonist administration on IκBκ degradation within the kidney under normal conditions and during LPS-induced AKI. Nicotine treatment alone (in the absence of LPS) slightly enhanced renal IκBα levels by 10±4.9% (mean±SD) (not significant); whereas treatment with GTS-21 alone significantly increased renal IκBα expression by 33±8.9% (P<0.05) when compared to saline-treatment alone. LPS-induced AKI was accompanied by renal IκBα degradation which was significantly reduced by treatment of mice with nicotinic agonists (Fig. 5A). Consistent with these observations, LPS treatment significantly enhanced NFκB activation within the kidney which was attenuated by treating the mice with nicotinic agonists (Fig. 5B).

### Proteasome Inhibition Suppresses Inflammatory Mediator Production by Human Kidney Cells

**Figure 6:**
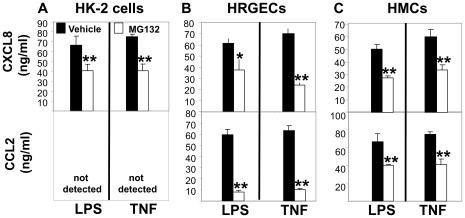
Proteasome inhibition blunts inflammatory responses by human kidney cell populations. (A) Human HK-2 renal tubular epithelial cells, (B) human renal glomerular endothelial cells (HRGECs), and (C) human mesangial cells (HMCs) were treated with either vehicle (filled bars) or proteasome inhibitor MG132 (10 µM, open bars) and then either LPS (1 µg/ml), or TNFα (20 ng/ml). Cell-free supernatants were analyzed for CXCL8 (upper panel) or CCL2 (lower panel) by ELISA 18 hrs later. Data are shown as cytokine levels (pg/ml) (mean±SEM) of 3 independent experiments. *p<0.05, **p<0.001 vs. LPS- or TNFα-treated (vehicle) cells.

Because NFκB-mediated inflammation is regulated via IκBα degradation by the ubiquitin-proteasome system, we investigated the effect of a widely used proteasome inhibitor, MG132, on LPS- and TNFα-induced chemokine production by various human kidney cells. Renal epithelial cells, glomerular endothelial cells and mesangial cells were treated with either vehicle or MG132 (10 µM) prior to LPS (1 µg/ml) or TNFα (10 ng/ml) addition. After 18 hours, cell supernatants were assayed for CXCL8 and CCL2 (Fig. 6). Similar to nicotinic agonists and the NFκB inhibitor, Bay11, MG132 treatment effectively inhibited LPS- and TNFα-stimulated chemokine production by all three human kidney cells (Fig. 6).

### LPS-AKI is Accompanied by Enhanced Kidney Proteasome Activity which can be Reversed by Nicotinic Agonists

**Figure 7:**
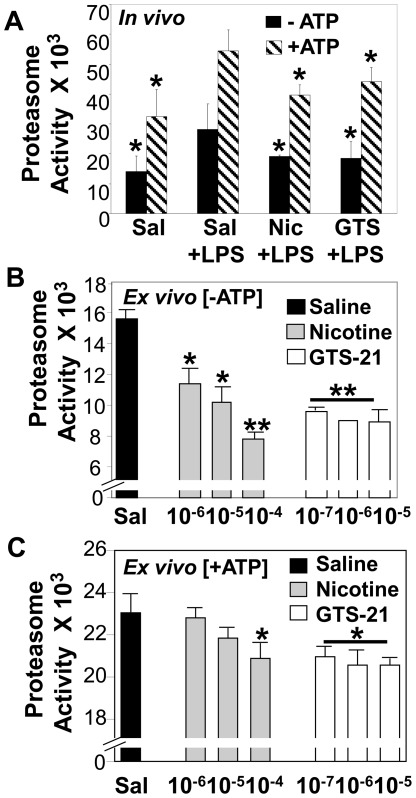
Septic AKI is characterized by dysregulated renal proteasome activity which is attenuated by nicotinic agonists. (A) Nicotinic agonist treatments vs. saline+LPS-treatment *in vivo*: Using the LPS-AKI model, mice were treated with saline, nicotine (1 mg/kg, i.p.) or GTS-21 (4 mg/kg, i.p.) (n = 5 per group); 1 hr post saline or LPS treatment, the effect of saline vs. nicotinic agonist treatment on LPS-induced renal proteasome activity (±ATP, 38 µM) was determined. *p<0.05 vs. Sal+LPS. (B-C) Nicotinic agonist treatments vs. saline treatment *ex vivo*: The effects of nicotine and GTS-21 (at the indicated molar concentrations) vs. saline treatment on proteasome activity in the absence of exogenously added ATP (B) and in the presence of exogenously added ATP (C) were assessed *ex vivo* using mouse kidney homogenates. Each experiment was repeated and the data are shown as proteasome activity (mean±SD). *p<0.05 and **p<0.01 vs. Sal (vehicle control).

Nuclear NFκB translocation leading to the production of inflammatory mediators depends, in part, on the degradation of phosphorylated IκBαby the ubiquitin-proteasome system. Because LPS-AKI is characterized by renal NFκB activation, we compared the proteasome activity of kidney tissues obtained from LPS-AKI mice vs. saline-treated (control) mice. LPS-AKI mice exhibited significantly enhanced renal proteasome activity compared to saline control mice (Fig. 7A). Using the septic AKI model, treatment with either nicotine or GTS-21 significantly attenuated both ATP-independent and ATP-dependent renal proteasome activity induced by LPS *in vivo* (Fig. 7A). Next, we examined the effect of exogenously added nicotinic agonists on renal proteasome activity *ex vivo* using renal homogenates. The addition of nicotine (10^−6^ M–10^−4^ M) or GTS-21 (10^−7^ M–10^−5^ M) significantly inhibited renal proteasome activity *ex vivo* in the absence (Fig. 7B) and presence of ATP (Fig. 7C), supporting a direct inhibitory effect of nicotinic agonists on renal proteasome activity.

## Discussion

The prevalence of AKI during sepsis (septic-AKI) significantly increases during the progression of sepsis with 19%, 23% and 51% observed among patients with moderate sepsis, severe sepsis, and septic shock, respectively (Reviewed in [1]). Among hospitalized patients, the mortality in patients with AKI complicating severe sepsis is an abysmal 70% compared to 39% in patients with non-septic AKI [26]. Unfortunately, there is no treatment for AKI or septic-AKI other than supportive care and treatment of the underlying disorders. Because nicotinic agonists exert beneficial anti-inflammatory activity [18]–[22], we examined the effects of nicotinic agonist treatment during experimental LPS-induced septic AKI. We found that administration of nicotine or GTS-21 during LPS-induced AKI significantly reduced the infiltration of leukocytes into the kidneys and reduced BUN levels (suggestive of reduced kidney injury) (Fig. 1). Using the LPS-AKI model and various human kidney cells we explored the mechanisms by which nicotinic agonists attenuate septic AKI.

Both LPS and TNFα have been found in the glomeruli of rats following LPS administration [10], [13]. LPS-induced AKI is characterized by renal damage mediated, in part, through localized renal TNFα production [6], [14] and enhanced circulating TNFα [27]. TNFα is produced by isolated glomeruli following treatment with LPS [13], as well as several kidney cell types (e.g. mesangial and tubular epithelial cells) [11], [12]. Local renal TNFα production promotes cytotoxic and inflammatory responses within the kidneys that trigger the generation of additional cytotoxic and chemotactic mediators. Like TNFα, both CCL2 and CXCL10 have been implicated in renal inflammation. Localized CCL2 production activates tubular epithelial cells to promote tubulointerstitial damage and recruits monocytes which further amplify renal inflammation [28]. CXCL10 is chemotactic for T cells (which have been found to be important mediators of renal injury [29], [30]) and promotes T cell-endothelial cell interactions [31]. Using the LPS-AKI model, we found that administration of nicotinic agonists blunted kidney inflammation (e.g. TNFα, CCL2, and CXCL10, Fig. 2). To explore which kidney cell types might be targets of nicotinic agonists, we treated human renal tubular epithelial, glomerular endothelial, and mesangial cells with nicotinic agonists during LPS or TNFα stimulation *ex vivo*. HK-2 renal epithelial cells, which retain the phenotypic expression and functional characteristics of human proximal tubules [32], [33], produced significant amounts of CXCL8, but not CXCL10 or CCL2 following LPS stimulation; LPS-induced CXCL8 was significantly reduced by treatment with nicotinic agonists (Fig. 3A). Likewise, TNFα induced CXCL8 and low levels of CCL2 production by HK-2 cells which were significantly blocked by nicotinic agonists (Fig. 3A). We found that nicotinic agonists also significantly suppressed pro-inflammatory chemokine production by primary human renal glomerular endothelial cells following LPS or TNFα stimulation (Fig. 3B). Mesangial cells are localized around the blood vessels of the kidney at the mesangium where they function like smooth muscle cells to regulate capillary blood flow. Treatment of mesangial cells with nicotinic agonists significantly suppressed both LPS- and TNFα-induced inflammatory chemokine production (Fig. 3C). Similar to previous studies linking the inhibition of NFκB activation to reduced leukocyte infiltration during sepsis and endotoxic shock [34], we observed that both TNFα- and LPS-stimulated chemokine production by renal cells was blocked by the NFκB inhibitor Bay 11 (Fig. 3).

NFκB activation within the kidneys during septic AKI has been reported [35], [36] and appears to play a critical role in the regulation of numerous pro-inflammatory mediators during infection and endotoxemia [25], as well as the promotion of tubular damage in LPS-induced AKI [36]. The IκBα inhibitory subunit of NFκB is regulated by the ubiquitin-proteasome system [37], the major non-lysosomal mechanism responsible for degrading regulatory cellular proteins, as well as misfolded, damaged, and/or unnecessary proteins following ubiquitin tagging [38]; [39]. In the absence of LPS, there was a slight increase in renal IκBα levels observed following the injection of nicotine alone and a significant increase in renal IκBα levels following the injection of GTS-21 alone, suggesting that nicotinic agonists might either increase renal IκBα expression or prevent its degradation. Nicotinic agonist administration reduced IκBα degradation within the kidneys and significantly suppressed renal NFκB activation *in vivo* (Fig. 5), supporting the concept that targeting the NFκB pathway will protect against kidney damage associated with septic-AKI, as previously described [40], [41]. While skeletal muscle biopsies obtained from hospitalized patients with sepsis and multiple organ failure exhibit increased proteasome activity compared to healthy controls [42], the assessment of kidney proteasome activity during septic AKI has not been reported. Treatment of several human kidney cells, including tubular epithelial cells, endothelial cells, and mesangial cells with a widely known proteasome inhibitor, MG132, suppressed both TNFα- or LPS-stimulated chemokine production (Fig. 6). In addition, we found that LPS-induced septic AKI was characterized by enhanced ATP-independent and ATP-dependent renal proteasome activity *in vivo* (Fig. 7A) and this activity was significantly reduced by nicotinic agonist administration *in vivo* (Fig. 7A). Likewise, renal proteasome activity (ATP-independent and ATP-dependent) was inhibited by exogenously added nicotine or GTS-21 (Fig. 7B–C). Thus, our data support the localized regulation of proteasome inhibition and inflammation by nicotinic agonists. These findings are consistent with previous findings revealing that nicotinic agonists suppressed local inflammation in the absence of an intact vagus nerve [18]. The inhibition of kidney proteasome activity *ex vivo* by nicotine and GTS-21 suggests that nicotinic agonists directly block proteasome activity. Based on the preservation of the IκBα-NFκB complex via proteasome inhibition and the subsequent blockade of NFκB-mediated inflammation, proteasome inhibitors have emerged as potential therapies for treating several inflammatory conditions, including adjuvant-induced arthritis [43], acute pancreatitis [44], hypercholesterolemia-associated renal disease [45], and endotoxemia lethality [46], [47].

Nicotinic acetylcholine receptors (nAChRs) are comprised of α1−α10, γ, δ, and ε subunits, and β1−4 subunits. Outside of the neuromuscular junction nAChRs form heteropentameric receptors comprised of α and β subunits, and homomeric non-β-receptors comprised of either α7 and α9 subunits [48], [49]. Previous studies by our group have described the constitutive expression of the α2, α3, α5, α7, α9, α10, β1, β2 and β4 nAChR subunits in rat kidney [50]. Using immunohistochemical staining methods, we showed that the rat kidneys express the α2, α3, and α7 nAChR subunits (with endothelial cells specifically expressing the α7nAChR) [50]. While nicotine can bind to: α2β2, α4β2, α3β2, α4β4, α2β4, α3β4, and α7 subunits, GTS-21, an α7 selective partial agonist, binds α7 and α4β2 nAChRs. However, GTS-21 appears to only activate the α7nAChRs [51]. In addition, GTS-21 has higher efficacy for rodent α7nAChRs than human α7nAChRs [52]. Based on our results, we evaluated the expression of the α7, α4, and β2 subunits in mouse kidneys and human renal cells. Mouse kidneys express mRNA transcripts for the α7, α4, and β2 nAChR subunits. In addition, we examined the expression of nAChR by the human kidney cells used in this study. While all three cell types expressed the α7, α4, and β2 nAChR subunits, both HK-2 cells (human proximal tubular epithelial cell line) and HRGECs expressed the highest level of α7nAChR (Fig. 4). From these results, we demonstrate the expression of the α7nAChR (a homopentameric receptor), but it is difficult to confirm expression of the α4β2nAChR by these cells because the α4 and β2 subunits can form heteropentameric nAChRs with various β subunits and α subunits, respectively. Treatment of HRGECs and HMCs with αBGT, an α7nAChR selective antagonist, prior to treatment with nicotinic agonists significantly blocked their inhibitory effects on TNF- and LPS-induced CCL2 production (Fig. 3D), supporting the role of α7nAChRs in mediating the anti-inflammatory effects of nicotine and GTS-21.

Proteasomes are very large protein complexes composed of a core of stacked rings (20 S proteasome) flanked by regulatory particles that facilitate protein entry into the ‘degradation chamber’ [53]. The complexity of proteasomes implies potentially multiple mechanisms for their functional regulation. Drews et al demonstrated that the regulation of cardiac proteasomes involved in left ventricular hypertrophy is linked to the desensitization of the beta adrenergic pathway [54]. Interestingly, previous studies support the negative regulation of proteolysis by the cholinergic neurotransmitter acetylcholine. Blockade of acetylcholine release increased protein degradation by proteasomes in *C. elegans* while stimulation of post-synaptic nAChRs via acetylcholine suppressed proteolysis [55]. Similarly, nicotine-mediated proteasome inhibition in the brain (*in vivo* and *ex vivo*) has been reported. Rezvani and co-workers found that nicotine treatment reduced proteasome activity (by directly interacting with the 20 S proteasome), thereby affecting synaptic plasticity [56]. Together, these data support the hypothesis that neural cholinergic inputs (e.g. acetylcholine) and nicotinic agonists regulate proteasome activity by interacting (either directly or indirectly) with one or more components of the proteasome complex. Based on our data supporting the role of α7nAChRs, we postulate that increased intracellular cAMP levels following α7nAChR ligation [57] augments I*κ*B*α* levels to effectively reduce NF*κ*B activation [58]–[60]. Consistent with this concept, our data show that in the kidney, nicotinic agonists increase the cytosolic availability I*κ*B*α* protein and attenuate NF*κ*B activation by inhibiting proteasomal degradation of I*κ*B*α*. Future studies will investigate the potential mechanism(s) whereby nicotinic agonists and acetylcholine produced by electrical stimulation of the vagus nerve lead to the inhibition of proteasome activity.

In summary, previous studies highlight the broad-based therapeutic targeting of inflammation using nicotinic agonists via the regulation of NFκB activation [18], [21] (and Fig. 5) and STAT3 activation [50], [61]. Herein we demonstrate the suppression of LPS- and TNFα-induced inflammatory mediator production by resident kidney cells by nicotinic agonists, enhanced renal proteasome activity during LPS-AKI, and the attenuation of renal injury in LPS-induced AKI following treatment with nicotinic agonists (which suppressed both ATP-dependent and ATP-independent proteasome activity). Together, these studies support targeting multiple pathways of inflammation and proteasome activity using nicotinic agonists to protect against renal inflammation and injury observed during septic AKI.

## Materials and Methods

### Antibodies and Reagents

Anti-IκBα and GAPDH antibodies were purchased from Cell Signaling Technology (Danvers, MA). The α7nAChR (319) and α4nAChR (286) monoclonal antibodies were purchased from Santa Cruz Biotechnology (Santa Cruz, CA), and the β2nAChR (270) monoclonal antibody was purchased from Covance (Princeton, NJ). LPS (*E. coli* 011:B5), nicotine, and ATP were purchased from Sigma-Aldrich (St. Louis, MO). αbungarotoxin (αBGT) was purchased from Tocris (Minneapolis, MN) and Bay 11-7082 (Bay 11), an NFκB inhibitor, and MG132, a proteasome inhibitor, were purchased from Calbiochem (San Diego, CA). GTS-21 was provided by Dr. Y Al-Abed (The Feinstein Institute); GTS-21 was synthesized as described previously by a reaction of anabaseine dihydrochloride with 2,4-dimethoxybenzaldehyde according to [62] and confirmed using NMR and LC-MS analyses. Suc-LLVY-AMC was purchased from Enzo Life Science (Farmingdale, NY). Lactacystin was purchased from Boston Biochemical (Cambridge, MA).

#### Cell cultures

HK-2 (human renal proximal epithelial tubule) cells, purchased from ATCC (Manassas, VA), were grown in DMEM 10%FBS, penicillin, streptomycin, and glutamine. Normal human mesangial cells (HMCs) (Sciencell, Carlsbad, CA) and human renal glomerular endothelial cells (HRGECs) (Sciencell) were grown according to the manufacturers’ recommendations. For activation assays, cells were grown to confluency in 96-well plates and treated with vehicle or nicotinic agonists (used at optimal doses determined from our previous studies [61] or NFκB inhibitor Bay 11 (25µM or MG132 (10 µM) for 30–45 min prior to stimulation with either LPS (1 µg/ml) or TNFα (20 ng/ml) (n = 3 per condition). After a 20 hr incubation period, cell-free supernatants were collected and assayed for CXCL8, CCL2, and/or CXCL10 levels by ELISA (R&D Systems). Each experiment was repeated twice and the combined data are shown as mean (±SEM). In a separate set of experiments, HRGECs and HMCs were incubated with αBGT (2 µM) for 30 min before the addition of either nicotine (10 µM, speckled bars), or GTS-21 (1 µM, hatched bars), followed by stimulation with either LPS (1 µg/ml, solid bar) or TNFα (20 ng/ml, solid bar) (n = 3 per condition). After an overnight stimulation, cell-free supernatants were analyzed for CCL2, as described above. Each experiment was repeated twice and the combined data are shown as mean %control (±SEM), with 100% being saline+LPS or saline+TNF. For the assessment of nAChR subunit expression, flow cytometry methods were used to detect the expression of specific nAChR subunits, as previously described [63]. Cultured cells were harvested using a non-enzymatic cell dissociation buffer (Cellstripper, Cellgro, Manassas, VA); all samples were blocked with 10% serum and then incubated with primary antibodies specific for α7nAChR (319), α4nAChR (286), and β2nAChR (270) subunits, according to the manufacturers’ directions. Antibody binding was revealed using biotinylated anti-rat IgM+IgG (SouthernBiotech, Birmingham, AL), followed by avidin-FITC (BD Biosciences). Rat Ig specific isotype controls (BD Biosciences) were used as negative controls and live fluorescent cells (without debris or aggregates) were analyzed on a FACSCalibur flow cytometer (BD). Data were analyzed using CellQuest data. Data are shown for each cell type as raw mean fluorescence intensity (FL) vs. counts and as the geometric mean fluorescence intensity or MFI (±SD) for each subunit for n = 3 samples.

### Animals

Animal protocols were reviewed and approved by the Institutional Animal Care and Use Committee (IACUC) of the Feinstein Institute for Medical Research prior to experimentation (IACUC#2005-049). All animals were housed, maintained, and manipulated according to the guidelines described in the National Research Council’s Guide for the Care and Use of Laboratory Animals.

#### Animal model of LPS-induced AKI

Swiss Webster mice (female, ages 8–10 wks, Taconic Farms, n = 5−8 per group) were injected with LPS (5 mg/kg, i.p.) immediately after saline (200 µl, i.p.), nicotine (1 mg/kg, i.p.), or GTS-21 (4 mg/kg, i.p.). These doses were based on our previously published studies [22]. Mice not euthanized prior to 6 hrs were treated with either additional saline (200 µl, i.p.), nicotine (1 mg/kg, i.p.), or GTS-21 (4 mg/kg, i.p.) at 6 and 18 hrs post LPS. Mice were euthanized by CO_2_ asphyxiation at indicated time points following the injection of LPS. Blood was collected immediately by cardiac puncture and serum was isolated and assessed for blood urea nitrogen (BUN) levels (measured by the Core Laboratories of the North Shore-LIJ Health System) or stored at −80°C. Cortical kidney samples were collected and either immediately flash-frozen in liquid nitrogen (cytokine and myeloperoxidase (MPO) assays) or immediately processed for the isolation of nuclear and cytoplasmic fractions for NFκB activation studies (see below).

### Assessment of Systemic and Renal Inflammation During LPS-induced AKI

Mouse serum TNFα levels were assessed at 2 hr post LPS injection and kidney cytokine/chemokine levels were assessed at 3 hrs post LPS. Frozen cortical renal tissue specimens (200 µg) were homogenized in 200 µl lysis buffer (Tris buffered saline pH 7.3 containing 0.5%TritonX-100 and protease inhibitor cocktail) on ice using a Dounce homogenizer; supernatants were collected after centrifugation. Serum and homogenates (n = 5−8 mice per group) were assayed for cytokines and chemokines by ELISA and protein levels by BioRad protein assay (assays performed in triplicate). Data are expressed as mean concentrations adjusted for protein levels.

### Analysis of Renal Leukocyte Infiltration During LPS-induced AKI

Mice were euthanized 3 hr post saline or LPS (in the presence or absence of nicotinic agonists) for assessment of leukocyte influx (n = 5−8 per group). Frozen cortical kidney specimens were assessed for leukocyte infiltration using an ELISA assay for mouse myeloperoxidase (MPO), according to the manufacturer’s directions (Cell Sciences, Canton, MA). Briefly, kidney samples (20 mg) were homogenized on ice in 200 µl lysis buffer (200 mM NaCl, 5 mM EDTA, 10 mM Tris pH 7.4, 10% glycerin, and protease inhibitor cocktail). After two centrifugation steps, lysates (1∶4 dilution) were assessed for MPO levels by ELISA, using a standard curve ranging from 0–500 ng/ml.

### IκBα Assessment and NFκB Activation Studies

Nuclear and cytoplasmic extracts were isolated from fresh cortical kidney specimens (n = 5−8 mice per group) collected 3 hrs post saline or LPS treatment (in the presence and absence of nicotinic agonist administration) using a nuclear extraction kit (Active Motif, Carlsbad, CA). Nuclear extracts were analyzed for NFκB activation using the TransAM NFκB p65/NFκB p50 Chemi Transcription Factor Assay Kit (Active Motif), according to the manufacturer’s instructions. IκBα protein levels (and GAPDH levels) in the cytoplasmic extracts were determined by Western blotting using specific anti-IκBα and GAPDH antibodies (a representative blot is shown). The density of the bands was determined using the NIH image program and the ratios of IκBα:GAPDH densities are shown.

### Assessment of nAChR mRNA Expression by Mouse Kidney Tissue Using QPCR

RNA was isolated from 100 mg of normal mouse kidney tissue using the RNeasy® Mini kit (Qiagen, Valencia CA) with DNase treatment, according to the manufacturer’s directions. The purity/concentration of total RNA was assayed using the NanoDrop spectrophotometer (Wilmington, DE). QPCR was used to assess specific nAChR mRNA expression using the following primers (and Roche Universal library probes): α7nAChR: forward: 5′-ccctggctttgctggtatt-3′, reverse: 5′-gcatgaagacagtcagagaaagtaa-3′ (probe 27);α4nAChR: forward: 5′-cgtccagtacattgcagacc-3′, reverse: 5′-atggccacgtatttccagtc-3′ (probe 29); β2 nAChR: forward: 5′-actctatggcgctgctgttc-3′, reverse: 5′-tcctctgtgtcagtacccaaaa-3′ (probe 76); HPRT1 forward: 5′-tcctcctcagaccgctttt-3′, reverse: 5′-cctggttcatcatcgctaatc-3′ (probe 95). Briefly, PCR reactions were performed in quadruplicate using the Eurogentec One step RT qPCR mastermix, 100 ng RNA and the Roche 480 Light Cycler using the following conditions: 48°C for 30 min, 95°C for 10 min followed by 45 cycles of 95°C for 15 secs and 60°C for 1 min. Relative gene expression was calculated using the comparative Ct (ΔΔCt) method as target gene to HPRT1 (housekeeping gene) for normalizing transcript levels [64].

### Renal Proteasome Activity Assays

The effect of nicotinic agonists on kidney proteasome activity was determined using the Suc-LLVY-AMC (chymotrypsin-like) substrate in the absence and presence of exogenously added ATP, as previously described [65]; [66]. For *in vivo* proteasome inhibition assays, mice (5 per group) were treated with saline, GTS-21 (4 mg/kg, i.p.) or nicotine (1 mg/kg, i.p.). Cortical kidney sections were isolated from mice, flash-frozen, and stored at −80°C; frozen kidneys were homogenized in freshly prepared assay buffer (50 mM HEPES, pH 7.5, 20 mM KCl, 5 mM MgCl_2_, 1 mM DTT) and used immediately. Briefly, 5 µg kidney homogenates (per well, with each sample run in triplicate) was incubated with vehicle or lactacystin (15 µM) for 20 min at RT prior to addition of assay buffer containing either 0 or 38 µM ATP. The reaction was initiated with the addition of substrate (Suc-LLVY-AMC) and incubated for 30 min at 37°C. This concentration of ATP (38 µM) was chosen because it produced maximal stimulation of proteasome activity using mouse renal homogenates (5 µg). The assay was terminated by the addition of 150 µl cold ethanol and read in a VictorX3 fluorescence plate reader (Perkin Elmer, Ex 360/Ex440 nm). Proteasome activity (chymotrypsin-like activity in fluorescence units) produced (±exogenously added ATP) was derived by subtracting the level of fluorescence obtained within 30 min in the presence of lactacystin from the values obtained without lactacystin after normalization. For the *ex vivo* assays, cortical kidney homogenates (5 µg) were incubated with nicotinic agonists (as indicated) or lactacystin (15 µM) for 20 min at RT prior to the addition of ATP (or no ATP) and substrate, as described above. The data from three separate experiments were averaged and results are shown as the mean proteasome activity (±SD).

### Statistics

Data are expressed as mean±SD (or ±SEM), as indicated. For data with multiple comparisons, data were analyzed by one way ANOVA followed by the Dunnett’s test (to compare each treatment to control value). When only two samples were compared, Students t tests were used for single comparisons between treatment and control. P values <0.05 were considered significant.
